# Etiologies of Acute Bronchiolitis in Children at Risk for Asthma, with Emphasis on the Human Rhinovirus Genotyping Protocol

**DOI:** 10.3390/jcm12123909

**Published:** 2023-06-08

**Authors:** Ahmad R. Alsayed, Anas Abed, Mahmoud Abu-Samak, Farhan Alshammari, Bushra Alshammari

**Affiliations:** 1 Department of Clinical Pharmacy and Therapeutics, Applied Science Private University, Amman 11931, Jordan; 2 Pharmacological and Diagnostic Research Centre, Faculty of Pharmacy, Al-Ahliyya Amman University, Amman 11931, Jordan; 3 Department of Pharmaceutics, College of Pharmacy, University of Hail, Hail 2440, Saudi Arabia; 4 Department of Medical Surgical Nursing, College of Nursing, University of Hail, Hail 2440, Saudi Arabia

**Keywords:** acute bronchiolitis, asthma, genotyping, human rhinovirus, sequencing, virus, bioinformatics

## Abstract

This research aims to determine acute bronchiolitis’ causative virus(es) and establish a viable protocol to classify the Human Rhinovirus (HRV) species. During 2021–2022, we included children 1–24 months of age with acute bronchiolitis at risk for asthma. The nasopharyngeal samples were taken and subjected to a quantitative polymerase chain reaction (qPCR) in a viral panel. For HRV-positive samples, a high-throughput assay was applied, directing the VP4/VP2 and VP3/VP1 regions to confirm species. BLAST searching, phylogenetic analysis, and sequence divergence took place to identify the degree to which these regions were appropriate for identifying and differentiating HRV. HRV ranked second, following RSV, as the etiology of acute bronchiolitis in children. The conclusion of the investigation of all available data in this study distributed sequences into 7 HRV-A, 1 HRV-B, and 7 HRV-C types based on the VP4/VP2 and VP3/VP1 sequences. The nucleotide divergence between the clinical samples and the corresponding reference strains was lower in the VP4/VP2 region than in the VP3/VP1 region. The results demonstrated the potential utility of the VP4/VP2 region and the VP3/VP1 region for differentiating HRV genotypes. Confirmatory outcomes were yielded, indicating how nested and semi-nested PCR can establish practical ways to facilitate HRV sequencing and genotyping.

## 1. Introduction

Due to wheezing, viral respiratory infections are the leading cause of hospitalization among newborns and young children [1]. The most common cause is thought to be respiratory syncytial virus (RSV). Other infections, in addition to *Mycoplasma pneumoniae*, cause wheezing in children, including human metapneumovirus (hMPV), influenza virus, parainfluenza virus, and adenovirus [2,3,4,5]. However, human rhinovirus (HRV) has been identified equally frequently as RSV in children hospitalized with wheezing disorders [6,7,8,9,10,11]. Several studies indicate that HRV is a substantial risk factor for later wheeze or asthma development [12,13,14,15,16].

The family “Picornaviridae”, genus “Enterovirus” (EV), has three HRV species: HRV-A, -B, and -C. Over 160 rhinovirus types have been identified, which include 99 classic HRV-A and HRV-B types [17], in addition to six novel HRV-A genotypes, five novel HRV-B genotypes, and fifty-one novel HRV-C genotypes. 

Screening methods based on polymerase chain reaction (PCR) are less complicated and more objective [18]. A classification system based on sequence data was optimal because PCR is a ubiquitous technique in laboratories and online databases contain a wealth of nucleotide sequence data. Such a system was devised for EV in 1999 [19]. Owing to their sensitive nature, it is possible to facilitate the diagnosis of previously unencountered HRV variants [20,21,22,23] and prototypic strains [24,25,26]. Noteworthy, sequence motifs of this kind constitute the aim of almost all primers and probes that have undergone publication, but it is important to recognize that they each reflect a distinct placement, length, and assay type (the standard, quantitative, nested, and one-step PCR approaches are employed).

The application of the HRV’s 5′ untranslated region (UTR) for genotyping purposes does reflect a variety of limitations. As a case in point, the 5′ UTR is linked to the non-specific amplification of RNA large regulator non-coding RNA B2 or human genomic DNA chromosome 6 sequences, both of which give rise to a 424 bp non-specific product (similar in size to the virus-specific amplicon, 390 bp) [23,27,28]. It is expected for non-specific results of this kind to arise in the context of clinical samples reflecting high human RNA concentrations or clinical samples that have been contaminated with genomic DNA. Owing to the suggested recombination between species, aspects of the 5′ UTR sequences for the HRV-A and C types are characterized by a certain degree of genetic similarity [29,30]. Furthermore, structural genes, as a result of considerable sequence variance, cannot be viably employed as a universal diagnostic primer. Nevertheless, as identified with a phylogenetic analysis of the capsid-coding regions, the A, B, and C HRV species are clearly delineated, and the disparities that exist between each mean that the regions can be distinguished from one another [31,32,33,34].

It was necessary to consider the probable molecular genotype determinants to classify EV based on sequence data. Studies of the viral-capsid protein 1 (VP 1) sequence divergence led to the proposal of a 25% nucleotide and 12% amino acid divergence threshold for classifying EV types [19]. Numerous new varieties of EVs have been classified based on these thresholds [35,36,37,38,39]. As some EV isolates have documented evidence of recombination within the capsid region [40,41], it is recommended that only VP1 be used for typing in EV. Previous efforts to classify EV by VP2 sequences have been unsuccessful [42,43].

In standard practice, neutralization assays have been superseded by molecular methods for classifying EV isolates. It has been determined that these methods consistently outperform serotyping in terms of accuracy, efficiency, and classification of new types [44].

It is no longer practicable or desirable to consider all HRV types as a single biological and clinical entity, as their genetic diversity and range of distinct clinical manifestations are so extensive. There are currently no conclusive links between any HRV type and a specific disease. As the spectrum of severe clinical illnesses attributed to HRV infection becomes better understood, it will likely become necessary to routinely screen for HRV in diagnostic settings and initiate large-scale epidemiological studies to determine circulation patterns and strain associations. A simple and practical system of classifying HRV into types, analogous to the system currently used for EV [45], will facilitate the investigation of potential outbreaks and nosocomial transmission as well as type-specific biological properties, such as the identification of types with potentially increased virulence.

The immediate aim of this research has been to establish a viable protocol by which it is possible to classify HRV species and types and to ensure that this protocol is characterized by high sensitivity, specificity, and cost-effectiveness. There are no epidemiologic investigations in Jordan on the prevalence of the causative pathogens in young children with acute bronchiolitis, focusing on HRV genotypes. Therefore, we used reverse-transcriptase polymerase chain reaction (RT-PCR) testing on the nasopharyngeal secretion of young children brought to the emergency room (ER) for the first time for acute wheezing to identify the related pathogen(s).

## 2. Materials and Methods

### 2.1. Study Design and Population

The approval of the study was received from the Research Ethics Committee of the Al-Rayhan Medical Center, Amman, Jordan (ARMC-2021-IRB-7-1), with informed, written consent from the parents of the children. 

Between August 2021 and August 2022, we invited the participation of children between 1 and 24 months of age admitted to the ER with the first episode of acute wheezing associated with a viral upper respiratory infection. 

We excluded children with (1) symptoms for longer than seven days, (2) previous endotracheal intubation, (3) a history of asthma or atopy with an excellent response to the first dose of β2-agonist nebulization, (4) a contraindication to corticosteroid, (5) premature birth, or (6) current or a history of COVID-19. 

### 2.2. Data and Specimens’ Collection and Follow-Up

A sample of nasopharyngeal secretion was collected from each child upon admission to the ER by inserting a disposable suction catheter connected to a mucus extractor and applying gentle suction without inserting any solution into the nostrils. The obtained secretion was promptly placed in a tube containing viral transport media and sent to a laboratory, where samples for pathogen detection were aliquoted and frozen at −80 degrees Celsius until processing. The specimens were deposited in 2 mL cryo-safe-labeled tubes containing the laboratory’s identification and the date. 

Each child’s demographic characteristics, medical history, treatments, and clinical course were thoroughly recorded. All children were offered optional follow-up consultations one month after discharge, then every three to six months for three years. Children diagnosed with respiratory disease or asthma were welcome to return for longer-term, routine follow-ups. All medical records will be examined at the end of the third year. Any child who misses a follow-up appointment will be contacted by phone to inquire about any respiratory diseases and treatments they may have received.

### 2.3. Nucleic Acid Extraction from Samples

The samples were thawed in a water bath at 37 °C before nucleic acid extraction. The automated total nucleic acid extraction was performed by the BIOBASE Kit from 200 μL samples using the magnetic beads method (Biobase Biodustry (Shandong) Co. Ltd., Shandong, China) [46,47]. After extraction, the 100 μL eluted nucleic acid samples were used for virus detection. Then, the remaining volume of samples was transferred to 1.5 mL conical tubes and deeply frozen at −80 °C for HRV retrospective investigation at the end of the study. 

### 2.4. Respiratory Viral Detection

The respiratory samples were subjected to real-time qPCR testing (amplification mix preparation) in a respiratory viral panel, including Adenovirus (ADV), Bocavirus (BOV); Coronavirus (COV); Generic influenza (FLU); Human rhinovirus (HRV); Metapneumovirus (MPV); Parainfluenza (PF); and Respiratory syncytial virus (RSV), covering several sub-types as detailed in a recent study [48]. The TIANLONG: Real-Time PCR System with 48-well block equipment was used using the appropriate kits and primers, according to a recent publication [48]. HRV detection in those clinical specimens was carried out using specific primers that targeted the HRV genome’s highly conserved 5′-NCR. According to a recent study, the absolute quantification approach was utilized to analyze the samples [49]. SARS-CoV-2 detection was performed using real-time PCR, as outlined in previous studies [49,50].

### 2.5. PCR Protocol for HRV-Positive Samples Targeting VP4/VP2 and VP3/VP1 Regions

#### 2.5.1. PCR Primers for HRV VP Regions

Different PCR primers were used to cover various genome regions in the viral-capsid protein (VP) of HRV. All PCR primers were designed in previous studies (Table 1) and synthesized by Sigma-Aldrich, Gillingham, UK, using DST purification (the “stage A” of PCR optimization in this study, as shown in Figure S1) and HPLC purification (stage B of this study). These primers target VP1, VP3/VP1, the VP4 region, and a section of the VP2 genome region (VP4/VP2). As recommended by Sigma, the primers were reconstituted in the corresponding volumes of nuclease-free water to obtain a concentration of 100 µM. After 30 min, the tubes were vortexed for a few seconds. A quantity of 10 µM is prepared for each primer by diluting 20 µL of the original primers’ tubes with 180 µL nuclease-free water. All primers were stored in a −20° freezer.

The term “serotype” indicates identification and classification by directly investigating antigenic properties. Therefore, we have used the term “genotype” or simply “type” to represent HRV types identified and classified by sequence data alone. The highly conserved “5′ untranslated region” (5′-UTR), and “5′ non-coding region” (5′-NCR) are used interchangeably throughout the paper.

#### 2.5.2. Pre-PCR and PCR Protocol

Different master mixes were used with different primer combinations, as shown in Table S2. The second-round PCR used the D, E, X, and Z master mixes to increase the specificity of detection.

This study was divided into two main stages: “stage A” which is limited in the flow chart (Figure S1), briefly included ten steps of PCR optimization using DST-purified primers. Whereas “stage B” represents the second period and most of this method development study.

Regarding stage B, initially, the first-round PCR’ master mix, which contained reverse transcriptase (RT) reagents from SuperScript™ III Platinum™ One-Step RT-PCR Kit (Invitrogen, Thermo Fisher, Oxford, UK), was processed following the manufacturer’s instructions, and 20 µL aliquots were placed into the reaction tubes (Table S3). Following this, 5 µL of the RNA extraction eluate from the clinical samples was added to the reaction tubes containing the first-round PCR master mix to create a final volume of 25 µL before transferring the reaction mixture to the PCR machine.

The LightCycler^®^ Multiplex DNA master (Roche, Burgess Hill, UK, cat. no. 07 339 577 001) and the Platinum^®^ SYBR^®^ Green qPCR SuperMix-UDG (Thermo Fisher, UK, cat. no. 11733038) were both used in the second-round PCR with the D, E, X, and Z master mixes (Tables S4 and S5, respectively). A master mix comparison was performed using the four highest HRV-load samples.

For HRV screening, DNA fragments were amplified via PCR. The PCR protocol involves a series of temperature-dependent stages performed cyclically. The reaction mixture was reverse-transcribed into cDNA. Then the mixture was subjected to denaturation of the target DNA (either cDNA or first-round PCR product), followed by primer annealing, nucleotide extension, and a final extension step. Table S6 represents the thermal cycles applied to all stage B’s first and second-round PCR reactions in this study. 

The DNA polymerase is rendered unreactive at room temperature by complexing the Platinum Taq with an activity-blocking antibody, thereby increasing sensitivity by halting non-specific annealing. Polymerase activity resumes at higher temperatures during thermal cycling (Table S6) as the complexed antibody is denatured.

The first-round product attained from the Superscript III reaction was used directly in the second round of PCR, as previously described by mixing 1 µL of this product with 24 µL of the second-round PCR’ master mix.

#### 2.5.3. Variations of the Standard PCR Technique (Nested and Semi-Nested PCR)

The study also adopted other PCR cycling profiles in addition to the standard PCR described. These were nested and semi-nested PCR. Table 1 shows the master mixes summary detailing the PCR products’ length and the primers used for semi-nested and nested PCR.

#### 2.5.4. Agarose Gel Electrophoresis

The gel electrophoresis used for stage B in this study is 1% agarose (ABgene Multi ABgarose, Thermo Fisher Scientific, UK). The agarose gels were dissolved in 1X Tris-Borate-EDTA (TBE) buffer (made from ×10 Gibco Ultra-Pure TBE buffer) (Thermo Fisher Scientific, UK) containing 1× pegGREEN. GeneRuler 1 kb or 100 bp DNA Ladder (Thermo Scientific, UK) was used. All PCR products were run at a constant voltage of 100 V for 1 h and 30 min. The resultant gels were observed under UV light and photographed using a BioSpectrumAC Imaging System V2.

#### 2.5.5. PCR Product Clean-Up Procedure

All PCR products underwent a PCR clean-up step to remove unincorporated primers and deoxyribonucleotide triphosphates (dNTPs) and improve sequencing reaction fidelity. The spin column PCR purification step was performed using the QIAquick PCR Purification Kit (Qiagen, Manchester, UK), combining three main phases: binding, washing, and eluting the DNA. The resulting mixture was then used directly for sequencing reactions.

### 2.6. Sanger Sequencing Workflow

**Figure 1:**
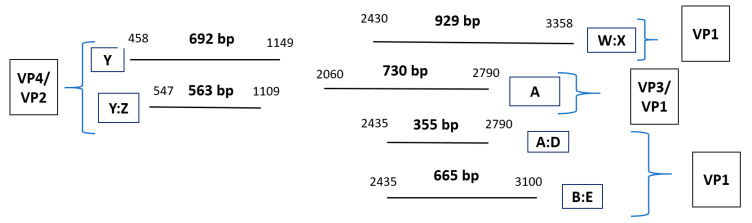
Illustration of polymerase chain reaction (PCR) products for the six optimal assays (with HRV genome targets’ regions). Above are given the first- and second-round PCR products (i.e., the horizontal lines) for the six optimal assays in the research, including the lengths, placements (in relation to one another), and the VP target regions in the HRV genome. Product length (bp) is indicated, and the PCR product numbers give the positional number of the nucleotides inside the HRV genome (i.e., the 5′ and the 3′, on the left and right, respectively). Abbreviations: viral-capsid protein (VP).

All HRV sequencing was performed using the Sanger method, in which the reaction mixture comprises all four-chain terminating dideoxynucleotides labeled with distinct fluorescent dyes, allowing the DNA sequence to be determined sequentially [18]. This was accomplished using the ABI BigDye Terminator kit (Applied Biosystems, Cheshire, UK), with the reagents and cycling conditions described in the supplementary data (Tables S7 and S8). Using appropriate PCR primers, PCR products were sequenced in both sense and antisense orientations to enable dual coverage (Figure 1). The quantity of BigDye used in each reaction was modified based on the fragment size.

A pGem and NTC were included as controls for each set of sequence reactions. The plate was sealed with an adhesive PCR seal (ABGene, AB-0558) and spun briefly (1500× *g* for 30 s) in a centrifuge to ensure the reaction mix was at the bottom of the well. The results of Sanger sequencing were returned in the form of FASTA and corresponding ABI files.

### 2.7. Bioinformatic Methods

#### 2.7.1. Sequence Alignment and Database Searching

All phylogenetic and evolutionary analyses depend on correctly identifying and aligning homologous sequences in a sequence alignment. The alignment process is fundamental to all subsequent analyses and prevents erroneous conclusions regarding phylogeny, genetic diversity, and recombination.

Sequences were imported into Lasergene SeqMan Pro version 15 (DNASTAR Inc., Madison, WI, USA) and initially aligned with the Pro Assembler algorithm implemented within the software. Any gaps and mismatches between the sense and antisense sequences were resolved by inspecting the associated chromatograms. Amino acid sequences were obtained by translating nucleotide sequences in the DNASTAR V15 software package using a standard genetic code.

Sequences of HRV PCR products were analyzed using nucleotide and protein BLAST (BLASTN and BLASTX, http://blast.ncbi.nlm.nih.gov/Blast.cgi, accessed on 1 November 2022) to obtain the maximum number of matching and potentially homologous sequences for analysis.

BLAST is a heuristic that, despite being rapid and relatively accurate, sacrifices some accuracy for speed and cannot guarantee that all homologous sequences will be returned. Consequently, optimizing particular parameters is essential for the endeavor’s accuracy.

An input query sequence in FASTA format is separated into “words” of a particular length. The entire database is then searched for occurrences of every possible word derived from the query sequence. These are 11 nucleotides in length by default. As a precise match of the entire word length is required for the algorithm to advance, the word length can be specified to modify the sensitivity and specificity of the protocol. When a match is found in a database sequence, the hit is extended by adding bases from the query sequence to both the 5′ and 3′ ends and searching for additional matches. The alignment is scored sequentially according to a specified match/mismatch penalty. The match/mismatch score’s default value is 1/−2. This was reduced to 1/−1 when searching for sequences with a greater dissimilarity. The alignment extension is continuously scored until its score falls below a predetermined threshold (20 for nucleotide sequences). The extension phase permits the distinction between meaningful and random matches. The returned sequences were allocated an expectation value (E-value), which estimates the probability that a hit is a false positive.

#### 2.7.2. Assembly of VP Region Sequences

**Figure 2:**
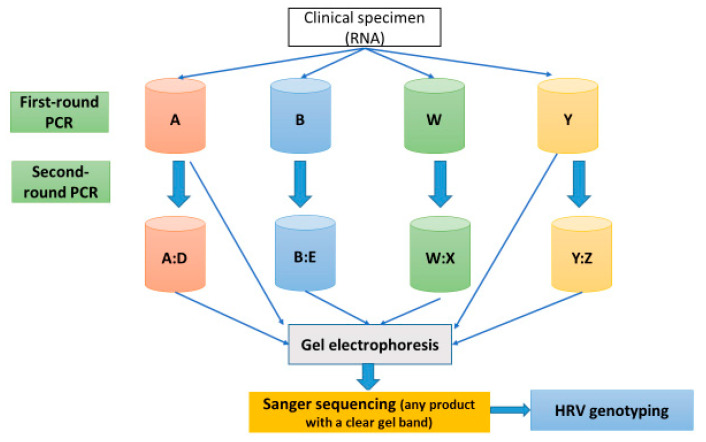
The workflow of HRV genotype analysis. A, B, W, and Y represent the first-round PCR assays (master mixes); A:D, B:E, W:X, and Y:Z are the second-round PCR assays (master mixes). Abbreviations: PCR: polymerase chain reaction; RNA: ribonucleic acid.

Sequence reads were downloaded, end-trimmed (to remove the primer sequence as well as low-quality sequences and base pairs), and assembled with the program SeqMan Pro version 15 (DNASTAR Inc., Madison, WI, USA). Pro Assembly algorithm parameters used for contigs assembly using SeqMan Pro are as follows: match size = 25, minimum match percentage = 70, minimum sequence length = 50, gap penalty = 0.00, gap length penalty = 0.00, match spacing = 150, and maximum mismatch end bases = 15. Additional manual inspections identified ambiguities or potential single nucleotide variants that were interrogated by further RT-PCRs and sequencing. To close gaps between assembled contigs, additional primer design and sequencing were investigated using SeqMan Pro and SeqBuilder version 15 (DNASTAR Inc., Madison, WI, USA) to enhance the sequence coverage. Figure 2 illustrates the analysis steps of HRV genotyping.

#### 2.7.3. Multiple Sequence Alignment, Nucleotide p-Distances, and Phylogenetic Analysis

The regions of the HRV genome analyzed in this study included the VP4/VP2 and VP3/VP1 of HRV-A, HRV-B, and HRV-C that are commonly used in studies of HRV molecular epidemiology [51,52,53,54,55] and downloaded from GenBank. Any highly diverse sequences from the particular multiple sequence alignments (MSA) were excluded. Pairwise sequence alignment and multiple sequence alignment (MSA) were performed using the software MegAlign version 15 (DNASTAR Inc., Madison, WI, USA).

Phylogenetic trees illustrate the relationships among the HRV genotypes of the study’s samples and the related references in the VP4/VP2 and VP3/VP1 regions. The input sequences were initially processed to produce the MSA and matrixes of identity and distances (divergences) between all sequence pairs. The unrooted phylogenetic trees were created using the Neighbor-Joining method [56] according to the distances (divergences) between all pairs of sequences in the MSA, with branch length proportional to the sequences’ divergence. The evolutionary distances were calculated using the Maximum Composite Likelihood method [57] in units of the number of base substitutions per site. The confidence of associated sequence (taxa) clustering was assessed by bootstrapping calculated from 500 replicates [58]. Bootstrap values of >70% indicate highly significant clustering, whereas values < 50% indicate that the clustering is statistically insignificant. Phylogenetic analyses were performed using MEGA7 [59]. Pairwise nucleotide p-distances were computed using the “Sequence Distances” program within the MEGA7 package. Divergence values were calculated as distance values × 100%.

#### 2.7.4. Recombination Analysis within the VP4/VP2 and VP3/VP1 Regions

The recombination predictions of the genomic sequences, aligned as described above, were conducted with a suite of programs within the RDP4 package version 3.2 [60]. The individual programs: RDP4 v3.2, Bootscan, Maximum X2, Chimaera, SiScan, and 3Seq were applied for the analysis.

Since no single program provides optimal performance under all conditions, any event supported by evidence from two or more analyses with *p*-values < 1.00 × 10^−5^ was considered a result consistent with recombination. Potential recombination events were also assessed by phylogenetic and alignment consistency examinations. Each program’s default settings were used, except as specified: Bootscan, number of bootstrap replicates, 500, window size, 100 bp, with step size, 10 bp; SiScan, window size, 100 bp, with step size, ten bp.

## 3. Results

### 3.1. Viral Detection

During the period of the study, 103 children were presented with acute wheezing linked to acute bronchiolitis. The parents of 93 (90.3%) of these children gave consent, making their children eligible for the collection of nasopharyngeal samples. A single specimen from each child on the same date was included to establish the rates of viral detection, and 91 samples were appropriately stored for viral detection. The total viral positivity rate was 79/91 (86.8%). The most frequently detected pathogen was RSV (42, 46.2%), followed by HRV (15, 16.5%), and FLU (13, 14.3%). Sixteen children (17.6%) were infected with dual viruses (Table 2). The baseline characteristics, clinical manifestations at enrolment, and clinical course for each of the children with any of the different etiologies were comparable (Table 3 and Table 4).

### 3.2. Real-Time qPCR for the Template Samples

Using real-time qPCR, the cycle threshold (C_t_) value range for the 15 tested respiratory specimens was 18.06 to 33.07. We used these samples’ four highest copy numbers (the four lowest C_t_ values) for PCR optimization.

### 3.3. HRV Detection Rate of First- and Second-Round PCR Assays Using Gel Electrophoresis

Lacking consistency was one of the features of the results from Stage A of the research (which was performed using the DST-purified primers) (Figure S1), and it was also the case that certain PCR products were non-specific. As for Stage B, combined with the negative control, 15 respiratory samples were analyzed by employing first- and second-round PCR assays. Figure S2, a gel picture, illustrates a first-round PCR product from a single sample, whereas Figure S3 illustrates the second-round products. Considering Figure S4, the suggestion is that the semi-nested and nested assays employed in this research could valuably aid the genotyping of HRV. For example, the gel-check outcomes of 10 assays for a pair of samples are given, with one characterized by an elevated C_t_ value (namely, 33.00). Noteworthy, several PCR products associated with specimens displaying a C_t_ value exceeding 30.00 were observed with respect to more than one assay, thereby emphasizing the effectiveness of this research’s assays.

We also compared the performance of the LightCycler^®^ Multiplex DNA master (Roche, UK, cat. no. 07339 577001) and the Platinum^®^ SYBR^®^ Green qPCR SuperMix-UDG (Thermo Fisher, UK, cat. no. 11733038). The Platinum^®^ SYBR^®^ Green qPCR SuperMix-UDG gave a better resolution on the gel for most of the tested PCR products. However, both gave the same positivity rate.

**Figure 3:**
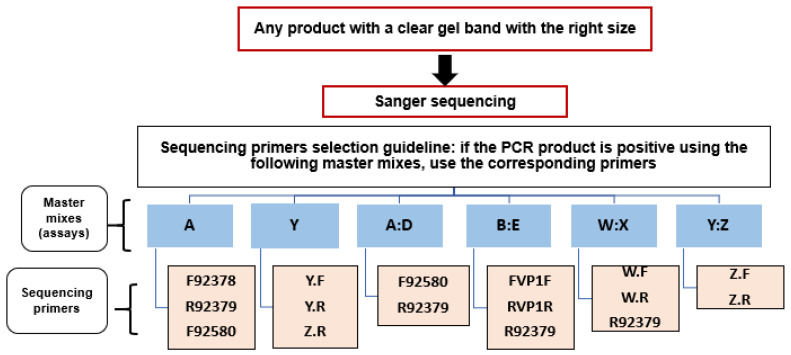
Sequencing primers’ selection guideline.

**Figure 4:**
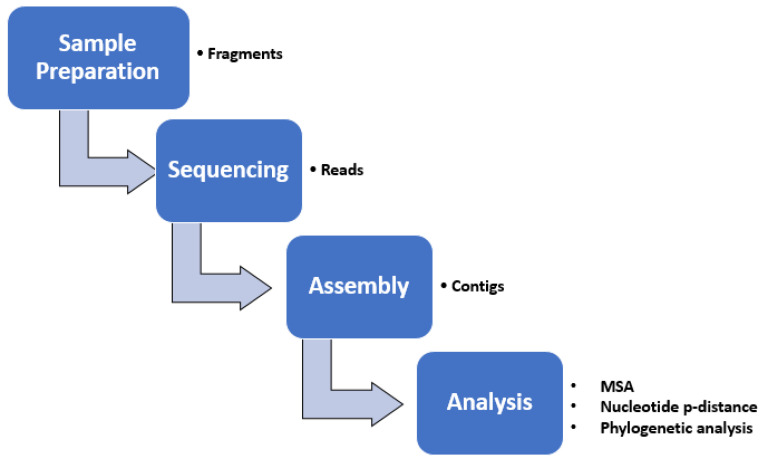
Summary of the analysis steps of HRV genotyping. The boxes represent the main steps, while the points beside the boxes summarize the output. MSA: multiple sequence alignment.

The degree to which assays (with various primers) successfully detected the viral capsid proteins (VPs) encoding genes is overviewed in Table S9. The level of positive results has been calculated by dividing the total number of positive samples by the total number of tested samples. Regarding PCR product positivity, the greatest detection rate among the second-round PCR assays was associated with the nested assay “Y:Z” (87%), which targeted the VP4/VP2 region. In turn, VP1 region assays, including the “A:D” semi-nested assay (40%), the “B:E” nested assay (30%), and the “W:X” nested assay (27%), followed this in terms of the next-highest level of positivity. The greatest detection rate among the first-round PCR assays was associated with “Y”, at a level of 53%; this assay targeted the VP4/VP2 region, followed by the “A” assay (40%), which targeted the VP3/VP1 region. Figure 3 illustrates the first and second PCR products for the optimal assays in this research, their lengths, and the VP target regions for the HRV genome. In Figure 4, the guidance workflow is given for sequencing all positive HRV respiratory specimens.

### 3.4. Sequencing and Sequence Analysis

Using the six assays with a total of 11 primers, sequencing reactions were successful in 95/119 reactions (80%) (Table S10). Lasergene SeqMan Pro version 15 (DNASTAR Inc., Madison, WI, USA) with the Pro Assembler algorithm implemented within the software created contigs in 8/15 samples (Table S11). Trim-ends of low-quality sequences and nucleotides were applied to end up with a mean/median quality (Q) of 24 (Table S12).

### 3.5. Pairwise Nucleotide p-Distances of HRVs

The distinction between intra-type and inter-type HRV was assisted by the inclusion of reference sequences of assigned genotypes, allowing the distributions of the intra- and inter-pairwise distances to be identified for VP4/VP2 (Figures S5 and S6) and VP3/VP1 (Figures S7 and S8) regions. Divergence values were calculated as distance values × 100%.

Among the strains detected in our study and the reference strains in the VP4/VP2 region, the p-distances (mean ± SD) for HRV-A, HRV-B, and HRV-C were 0.268 ± 0.062, 0.221 ± 0.036, and 0.336 ± 0.111, respectively (Figure S5A–C). Among our study strains and the VP3/VP1 region reference strains, the p-distances (mean ± SD) were 0.278 ± 0.066 for HRV-A and 0.518 ± 0.123 for HRV-C (Figure S6A,B).

The distributions of pairwise nucleotide p-distances were constructed. They showed a maximum within-species divergence of 44%, 31%, and 60%, respectively, for HRV-A, -B, and -C of the VP4/VP2 region (Figure S5), and 43% and 70%, respectively, for HRV-A and -C of the VP3/VP1 region (Figure S7).

When HRV sequences from all three species were compared, the pairwise nucleotide p-distances revealed that, as expected, the lowest values (15%) represented comparisons within the same type. In contrast, the highest (between 30% and 80% divergence) represented comparisons between isolates of different species. The vast number of comparisons ranging from 15% to 45% divergence indicate isolates of the same species but of various types. The minimum between-species divergence values for both regions were between 30% and 40% (Figures S5 and S7).

### 3.6. HRV Genotype Identification and Phylogenetic Analysis

**Figure 5:**
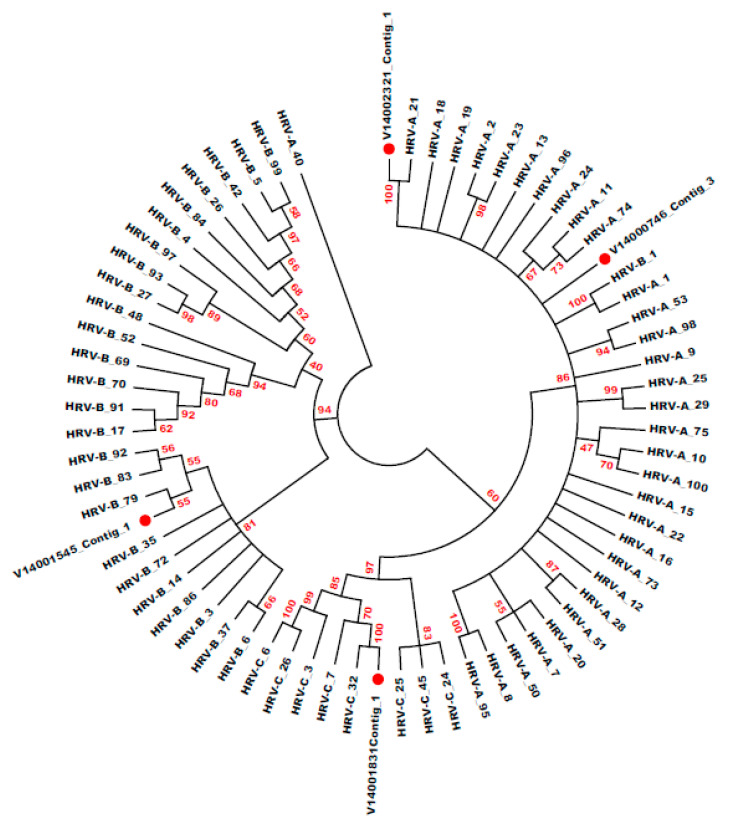
Phylogenetic analysis of HRVs based on the VP4/VP2 region nucleotide sequences (MSA 2). By employing the VP4/VP2 MSA 2 (545 nucleotides), it was possible to complete the neighbor-joining analysis by employing the maximum composite likelihood model. In addition, by employing bootstrapping computed using 500 replicates, it was possible to evaluate the confidence of the sequence clustering. In addition, the branches and those bootstrap values that exceeded 40% are given, and phylogenetic analyses were completed by employing MEGA7. HRV strains in this research are circled in red. The ideal tree (with the sum of the branch lengths = 7.964) is given. Seventy nucleotide sequences were incorporated into the analysis, and positions containing insufficient information, such as gaps, were removed. Therefore, the data set included 417 positions in total.

All HRV sequences were classified into groups based on bootstrap-supported phylogenetic clades that closely matched types assigned by sequence distances (Figure 5). Based on the phylogenetic tree reconstruction of 38 sequences, the 15 clinical isolates were assigned to seven different genotypes and strongly supported with highly significant bootstrap values (70–100%, Table S13 and Table 5). The data were bootstrap resampled 500 times to assess the robustness of the branches. Eight HRVs did not significantly match a specific genotype with >15% nucleotide divergence from the nearest reference HRVs. However, they had a high identity and very low E values with reference sequences in the corresponding region.

Some HRV-type sequences (4 HRV-A and 1 HRV-C) violated the VP1 threshold of 13% proposed in an earlier study [61] (Table 5 and Table S13). However, most sequences showed a minimum inter-clade VP divergence (with the nearest neighbor type group) greater than the proposed threshold (Figures S6 and S8).

### 3.7. Recombination Analysis within the VP4/VP2 and VP3/VP1 Regions

Analysis with the RDP software package highlighted no significant evidence of recombination between the study and reference strains (Table S14). RDP detection programs determine which sequence in the data set contributed the majority of the genome (referred to as the major parent) and which sequence contributed the minor or recombinant region (referred to as the minor parent). None of the recombinant sequences was detected by more than two programs, with average *p*-values of recombination events of <1.00 × 10^−5^.

## 4. Discussion

According to this study, HRV was the second-most common pathogen (after RSV) causing acute bronchiolitis in Jordanian children. As a result, HRV is an important risk factor for wheezing and acute bronchiolitis. HRV can infect the lower airway directly (it is not limited to the upper airway as previously thought), and multiple epidemiologic studies have shown that HRV is a primary source of lower respiratory infection in children [4,5,6,7,8,9,10,11,12,13,14,15]. This virus is responsible for many lower respiratory disorders, including pneumonia and asthma exacerbations [49,62,63].

As determined by molecular techniques, the prevalence of HRV in young children three years old with acute lower respiratory infections is between 17 and 35% [4,5,7,9,10,64,65]. This percentage was slightly higher than what we identified in our research. The age range and diagnoses of the research participants may account for the disparity. Acute bronchiolitis is a disease that primarily affects children under the age of two. Jartti et al. found that RSV was more prevalent in infants younger than one year, whereas HRV was more prevalent in older children [4].

Our results demonstrated that the variations in etiology, clinical presentation, and severity were not statistically significant. Children infected with HRV had a higher mean age and less fever than children infected with RSV or another virus. These data are comparable to those from Finland [8] and Thailand [66] but differ from those of France [5], which showed that RSV-infected children had significantly higher coughing and feeding difficulties and required more oxygen delivery than HRV-infected children.

Aeroallergen sensitization and virus-induced wheezing in childhood are recognized to be important risk factors for the development of asthma later in life, and children who have both risk factors are at an exceptionally high risk of acquiring asthma by the time they reach school age [11,14,67,68,69]. Prior to the discovery of HRV, the most prevalent infection causing wheeze and subsequent asthma was assumed to be RSV [70,71].

Recent studies have used cluster analysis to investigate the interplay between major bacterial species, their functions, and the host response to bronchiolitis in infants. This information, along with clinical, virus, and proteome data, was used to identify biologically distinct endotypes of bronchiolitis with differential risks of asthma development [72,73,74,75,76].

For the purpose of determining the degree to which the HRVs were genetically diverse, respiratory samples were applied in order to characterize the HRV variants in genetic terms. A range of primer combinations (including nested primers) were employed to achieve this, thereby efficiently amplifying the VP4/VP2, VP3/VP1, and VP1 regions of the HRV species and genotypes. Aside from Wisdom et al. [34], which investigated the role played by nested HRV detection assays, the literature about assay technique details is limited, thus motivating the present study.

The researchers understood that finding one assay with an HRV detection rate of 100% would be unlikely. This expectation stems from the recognition that the HRV genome’s regions interacted with each other throughout the study and were characterized by a high level of variance. In view of this, the optimal assays would be those that were operable for the greatest number of samples, and in an ideal case, those reflecting elevated C_t_ values.

The conclusion of the analysis of all available data in this study divided sequences into 7 HRV-A, 1 HRV-B, and 7 HRV-C types based on the VP4/VP2 and VP3/VP1 sequences. The nucleotide divergence between the clinical samples and the corresponding reference strains was lower in the VP4/VP2 region than in the VP3/VP1 region.

When HRV sequences from all three species were compared, the pairwise nucleotide p-distances revealed that, as expected, the lowest values were for comparisons within the same species or genotypes. At the same time, the largest (between 30% and 80% divergence) represented comparisons between isolates of different species. Many comparisons involving 15% to 45% divergence include isolates of the same species but different types. These findings show that HRV-C deviates slightly from the previously stated conclusion and warrants additional examination. A clear inter-species p-distance threshold supports the idea that HRV species can be characterized by nucleotide divergence in the capsid region. The most diverse strains are only represented by a few sequences in each case. As a result, it was difficult to identify whether these were variants that happened to be at the extreme end of the distribution of intra-type divergence values or variants that were midway through the process of diverging into a new HRV type. This may become obvious when more data from epidemiological and evolutionary investigations accrues.

Within EV isolates, recombination has been recognized within the capsid coding region [40]. However, recombination within the capsid coding region of HRV is believed to be relatively uncommon [77,78]. Our study’s analysis with the RDP software package highlighted no significant evidence of recombination between the study and reference strains. Due to dealing with short sequences (<2000 nucleotides), we cannot confirm that there is no significant recombination between the investigated regions.

### 4.1. Development of a Method for the Amplification of the VP Region of HRV

Performing genotyping directly from clinical specimens eliminates the necessity of cell culture passage and significantly decreases the time needed for an accurate diagnosis. This advancement has been successfully demonstrated in studies focusing on EV [79,80]. The assays developed in this research enable the amplification of specific regions of HRV’s VP from respiratory specimens, which typically have low viral concentrations. However, due to the extensive genetic variability, creating a single assay capable of simultaneously amplifying all three HRV species was not feasible. Even within a single HRV species, maintaining the necessary balance between primer degeneracy for amplifying all types and avoiding non-specific amplification posed a challenge.

When designing novel methods, PCR optimization is a fundamental stage. The individual reaction component concentrations were modified, including temperature and time parameters, which were brought inside the required range (thereby facilitating the adequate amplification of the desired DNA target sequences). Ultimately, this stemmed from the fact that applying one set of conditions for every PCR amplification is not practically possible.

Several studies have supported using a one-step PCR for EV typing, containing only the first round of amplification [81,82], proposing the idea that a “closed system” is less susceptible to contamination. Nevertheless, one of the aforementioned studies indicated that nearly one-third of the examined isolates were not efficiently amplified in the VP1 region, and the successfully amplified ones exhibited a notable presence of non-specific products [81]. Our findings demonstrate that implementing a nested PCR technique significantly decreases non-specific amplification, making it highly effective for amplifying samples with very low viral concentrations. Furthermore, contamination is notably minimized by strictly adhering to a “one-way” system in PCR laboratories, where different areas are spatially separated for nucleic acid extraction, first-round PCR, and second-round PCR.

Including the nested primers is a viable way to promote the degree to which DNA amplification is specific since it reduces the non-specific DNA products. Consequently, the second-round PCR product is not as long as the first. However, its amplification is more accurate since all amplification errors in the first round have a low probability of being replicated in the second PCR. Nevertheless, a key risk factor is the higher likelihood of non-specific contamination when applying the second-round PCR primers [79,83,84].

### 4.2. Proposed Criteria for HRV Genotyping

Given the well-conserved nature of 5′NCR, sequencing it for genotyping produces little valuable information. Contrastingly, those genes that encode the VPs are highly variable, and the sequences have a determinative impact on the genotype. Therefore, sequencing parts of these VP regions is expected to be useful for genotyping.

Using only capsid coding regions in type assignment should not detract from the value of continuous examination of other genomic regions, particularly where these regions may contribute to the phenotype and disease associations of HRV. In addition, using the VP1 region to identify novel HRV types should not impede the continued use of VP4/VP2 and 5′NCR screening protocols. Screening in these regions increases the likelihood of discovering previously unknown HRV types. In epidemiological studies and clinical associations, VP4/VP2 sequences can be used to identify the type. Recent and rapid accumulation of sequence data of the VP4/VP2 region on GenBank in contrast to sequences of the VP1 region, which are difficult to amplify without type-specific primers. If sequence data from the VP4/VP2 region are to be utilized in epidemiological or clinical studies, it is necessary to confirm that known HRV types can be accurately identified through analysis of this region. We would suggest the combined use of phylogenetic analysis and pairwise p-distance analysis in the classification of all HRV sequences. In cases where nucleotide divergence is insufficient to assign types definitively, phylogenetic relationships may be considered. The guidelines should be further reviewed as additional data becomes available.

Likely, less sizeable fragments of VP3/VP1 and VP4/VP2 are viable candidates for genotyping in HRV. In the study conducted by Oberste et al. [45,82], the researchers found that a 450-base segment at the 3′ end of VP1 can viably be applied for EV typing, and the outcomes were entirely correlated with the neutralization results. In another study, the researchers demonstrated a 100% correlation between a 303 nucleotide stretch of VP1 and neutralization data, but it should be noted that the research was only relevant for 59 GenBank strains [82]. Recently, pyrosequencing was proposed as a straightforward, rapid, and viable way to type EV [85].

### 4.3. The Importance and Implications of HRV Genotyping

Identifying HRV types by analyzing available sequence data within the capsid region can improve HRV genotyping, making the classification of all detected HRV sequences much more feasible. A global type of identification system should allow large-scale investigations of epidemiology, transmission, and evolution. Classification and centralized reporting of new HRV types improve the ability of researchers to study the distribution of new types quickly. Whereas previously, a considerable period of time would have been necessary while waiting for type-specific antiserum to be developed and dispatched, sequence data for comparison is almost instantly available worldwide.

HRV genotyping can be used to investigate the frequency and genetic diversity of HRV strains among respiratory samples referred for diagnostic testing and their relationship with different respiratory diseases.

Several of the current research’s findings could have vital implications. First and foremost, given that genetic variability has an adverse impact on the derivation of HRV vaccines and antiviral therapy, identifying those characterized by higher virulence might serve as a way to promote therapeutic advancement. In addition, despite how HRV infection has been implicated in the exacerbation of COPD and asthma, the information pertaining to the function of the various HRV species in impacting the exacerbation frequency phenotype is unknown. The clinical implications of HRV infection are much more severe than those associated with the common cold; HRV infection contributes to numerous effects, ranging from the absence of symptoms to COPD, pneumonia, and bronchiolitis. The complex nature of naturally occurring instances of COPD exacerbations is well documented. Yet, the information on the pathogenesis of HRV in the course of and following COPD episodes is limited. However, exacerbations are substantial events in COPD, and the possibility exists that HRV infection is associated with these exacerbations. Therefore, the possibility exists that information about the effect of HRV species on exacerbations could contribute to developing targeted therapeutic interventions, thereby counteracting the severity of the pulmonary diseases’ exacerbations.

### 4.4. Limitations and Future Research

The most important limitations of this study that need to be considered can be summarized as follows: First, the numbers of patients and samples were relatively small. Second, the inconsistencies in results are unfortunately unavoidable and should remain in place for clarity due to the nature of VP region variability, sequencing, or assembly errors.

As for the issue of further research, it would be helpful in subsequent studies to identify whether a particular threshold (one that divides pairwise p-distance comparisons into intra- and inter-type values) pertains to all HRV species. This could be achieved by establishing distributions of pairwise nucleotide p-distances using a more extensive data set. In addition, other researchers should consider replicating the present study to determine the degree to which the assay-related outcomes have yielded reliable conclusions. It would be valuable for some studies to employ larger samples. Finally, further research should examine the frequency and genetic diversity of HRV strains in the context of respiratory samples referred to for diagnostic testing and their connection with various respiratory diseases.

## 5. Conclusions

HRV is ranked second after RSV as the cause of acute bronchiolitis in children. The results of this study demonstrated the potential utility of the VP4/VP2 region in addition to the VP3/VP1 region for differentiating HRV genotypes. Confirmatory outcomes were yielded, indicating how nested and semi-nested PCR can establish practical ways to facilitate HRV sequencing and genotyping. Additionally, by employing a combination of first- and second-round PCR assays, optimal genome assembly can be achieved by targeting numerous HRV variable genome regions (namely the VP4/VP2, VP3/VP1, and the VP1 regions).

## Figures and Tables

**Table 1:** Summary of master mixes used for nested and semi-nested PCR.

1st-Round PCR Assay	OS Primer	OAS Primer	2nd-Round PCR Assay	IS Primer	IAS Primer	PCR Product Length (bp)
**Nested PCR**						
B	92,580 = F187	R 92383	E	F VP1F	R VP1R	665
W	W:F	W:R	X	X:F	X:R	929
Y	Y:F	Y:R	Z	Z:F	Z:R	563
**Semi-nested PCR**						
A	F 92378	R 92379 ^a^	D	F92580 = F187	R 92379 ^a^	355
C	F 92380 ^b^	R 92383	E	F VP1F ^b^	R VP1R	455

^a^ The reverse primer is the same in master mixes A and D. ^b^ The forward primer is the same in master mixes C and E. Throughout this study, the single letter, for example, A, B, C, and so forth, refers to assays (master mixes) used in a single-round PCR. Whereas a combination of 2 letters separated by a colon “:”, for example, A:D, Y:Z., and so forth, is used to refer to nested or semi-nested PCR assays, the first letter (the assay, the master mix) means that its first-round PCR product was used as a template with the second master mix (the second letter). These master mixes (assays) target various regions in the HRV genome; assay A targets VP3/VP1, whereas assays B, C, A:D, B:E, and C:E target VP1. Assays W, Y, W:X, and Y:Z are directing the VP4/VP2 region in HRV. Abbreviations: OS: outer sense, OAS: outer antisense, IS: inner sense, IAS: inner antisense.

**Table 2:** The frequency of the respiratory viruses detected in the study population (*n* = 91).

Aetiology	Total *n* (%)	Age, *n* (%)		
		1–6 Months *n* = 24	>6–12 Months *n* = 35	>12–24 Months *n* = 32
**Overall viral detection**	79 (86.8%)	17	32	30
ADV	3 (3.3%)	1	1	1
BOV	1 (1.1%)	0	0	1
COV	1 (1.1%)	0	0	1
FLU	13 (14.3%)	2	4	7
HRV	15 (16.5%)	3	4	8
MPV	3 (3.3%)	1	2	0
PF	1 (1.1%)	0	0	1
RSV	42 (46.2%)	10	21	11
**Overall dual detection**	16 (17.6%)	4	3	9
**RSV + HRV**	7 (7.7%)	1	1	5
**RSV + FLU**	4 (4.4%)	1	1	2
**HRV + FLU**	5 (5.5%)	2	1	2
**Not found**	12 (13.2%)	7	3	2

Abbreviations: “ADV: adenovirus; BOV: bocavirus; COV: coronavirus; FLU: influenza; MPV: metapneumovirus; PF: parainfluenza; HRV: human rhinovirus; RSV: Respiratory Syncytial Virus”.

**Table 3:** Baseline characteristics of the included children with the three common etiologies.

Characteristics	Total *n* = 79	RSV *n* = 42	HRV *n* = 15	FLU *n* = 13
Male, *n* (%)	45 (62.9)	23 (59.1)	8 (51.6)	9 (70.0)
Body weight (kg), Mean ± SD	8.30 ± 1.82	8.09 ± 1.82	8.52 ± 1.84	8.82 ± 1.86
Breastfeeding, *n* (%)	76 (94.1)	42 (92.7)	14 (96.8)	10 (93.3)
Duration of breastfeeding (months)	4	4	3	4
First relative atopy, *n* (%)	25 (31.6)	18 (20.9)	5 (19.4)	4 (26.7)

Abbreviations: “FLU: influenza; HRV: human rhinovirus; RSV: Respiratory Syncytial Virus”.

**Table 4:** Clinical features at presentation and clinical course of acute bronchiolitis.

Characteristics	Total *n* = 79	RSV *n* = 42	HRV *n* = 15	FLU *n* = 13
History of fever, *n* (%)	72 (91.1)	38 (90.5)	11 (73.4)	12 (92.3)
Temperature, °C Mean ± SD	38.8 ± 0.9	38.2 ± 0.9	37.9 ± 0.9	38.2 ± 0.8
95% CI	37.8–38.3	37.8–38.4	37.7–38.2	38.2–38.8
Duration of fever, days Mean ± SD	4.5 ± 3.0	4.4 ± 2.8	2.9 ± 2.9	4.5 ± 3.3
95% CI	3.9–4.7	3.8–4.8	1.8–3.4	3.2–5.7
SpO_2_ < 95%, *n* (%)	105 (61.8)	68 (61.8)	18 (58.1)	20 (66.7)
SpO_2_, % Min–Max	69–99	69–99	71–99	87–99
Mean ± SD	93.6 ± 4.7	93.7 ± 4.9	94.1 ± 4.8	94.0 ± 3.2
95% CI	93.1–94.4	92.8–94.6	92.3–95.9	92.8–95.2

Abbreviations: “FLU: influenza; HRV: human rhinovirus; RSV: Respiratory Syncytial Virus; SpO_2_: oxygen saturation”.

**Table 5:** Identification of HRV clinical isolates by BLASTN, BLASTX, phylogenetic tree construction, and p-distance.

Specimen ID	C_t_ Value	Genotype- BLASTN	E-Value	Genotype- BLASTX	E-Value	Genotype- Phylogeny	Bootstrap	Distance
V14002321	18.06	HRV-A_21	0	HRV-A_21	2.1 × 10^−117^	HRV-A_21	100	0.059
V14001831	18.10	HRV-C_32	0	HRV-C	1.8 × 10^−93^	HRV-C_32	100	0.047
V14001545	19.08	HRV-B	0	HRV-B	2.2 × 10^−94^	HRV-B_79	55	0.150
V14000746	19.12	HRV-A_40	0	HRV-A	2.3 × 10^−96^	HRV-A_40	96	0.055
V17004616	33.00	HRV-A	0	HRV-A	4.7 × 10^−31^	HRV-A_98	<50	0.173
V17004470	31.10	HRV-A	6.6 × 10^−52^	HRV-A	9.7 × 10^−19^	HRV-A_13	<50	0.236
V17005728	26.69	HRV-C	0	HRV-C	2.1 × 10^−86^	HRV-C_45	73	0.289
V17006129	31.90	HRV-C_7	0	HRV-C	1.4 × 10^−137^	HRV-C_7	99	0.098
V17006131	29.91	HRV-C	0	HRV-C	1.5 × 10^−96^	HRV-C_45	<50	0.296
V17006286	33.07	HRV-A	4.2 × 10^−69^	HRV-A	5.6 × 10^−21^	HRV-A_50	50	0.264
V17004189	28.88	HRV-A	2 × 10^−25^	HRV-A	5.2 × 10^−7^	NA	NA	NA
V17003665	30.09	HRV-C	1.7 × 10^−46^	HRV-C	2 × 10^−17^	HRV-C_24	69	0.268
V17004870	21.09	HRV-C	3 × 10^−20^	NA	NA	HRV-C_32	<50	0.927
V17005031	31.54	HRV-A	1.4 × 10^−121^	HRV-A	1.4 × 10^−53^	HRV-A_47	52	0.087
V17004381	28.75	HRV C	9 × 10^−66^	HRV C	7 × 10^−30^	HRV-C_36	100	0.066

## Data Availability

Data is unavailable due to privacy and ethical restrictions.

## Supplementary Materials

The following supporting information can be downloaded at: https://www.mdpi.com/article/10.3390/jcm12123909/s1, Table S1. Primers used for amplification and sequencing of the HRV region [33,43,44,86,87,88]. Figure S1. Stage A in PCR optimization. Table S2. Master mixes and primer combinations were used to detect the variable regions of the HRV genome. Table S3. The preparation of the first-round PCR master mixes. Table S4. The preparation of the second-round PCR reactions using the LightCycler^®^ Multiplex DNA master mix. Table S5. The preparation of the second-round PCR reactions using the Platinum^®^ SYBR^®^ Green qPCR SuperMix-UDG. Table S6. Thermal cycling conditions used in PCR reactions. Table S7. Reagents used in sequencing reactions. Table S8. Cycling conditions used in sequencing reactions. Figure S2. PCR product representation of master mix A. Figure S3. The second-round PCR product representation of A:D and C:E assays. Figure S4. Gel electrophoresis of first- and second-round PCR products from 10 different assays. Table S9. The PCR product positivity rate of HRV-VP assays using fifteen respiratory samples. Table S10. The successful representation of the Sanger sequencing primers. Table S11. A summary of gel-check, sequencing success, and sequence assembly results of the clinical HRV tested samples. Table S12. Description of the VP regions’ sequence assemblies. Figure S5. Distribution of nucleotide p-distances for HRV-A (A), HRV-B (B), and HRV-C (C) detected in the study compared with sequences from reference strains based on the nucleotide sequences of the VP4/VP2 region. Figure S6. Distribution of pairwise nucleotide p-distances for the VP4/VP2 region of all HRV sequences. Figure S7. Distribution of nucleotide p-distances for HRV-A (A) and HRV-C (B) detected in the study compared with sequences from reference strains based on the nucleotide sequences of the VP3/VP1 region. Figure S8. Distribution of pairwise nucleotide p-distances for the VP3/VP1 region of all HRV sequences. Table S13. Multiple sequence alignment (MSA) data and genotype identification. Table S14. A summary of recombination events detected in the multiple sequence alignments.
