# Supplementary material for: Etiologies of Acute Bronchiolitis in Children at Risk for Asthma, with Emphasis on the Human Rhinovirus Genotyping Protocol
Source: J Clin Med. 2023 Jun 8;12(12):3909. doi: 10.3390/jcm12123909 (PMC10299285; doi:10.3390/jcm12123909)
Supplement: Supplementary file 1 [file jcm-12-03909-s001.zip › jcm-2268779-supplementary.pdf]

TABLE S1. Primers used for amplification and sequencing of HRV region.

Region	Primer	Polarity	Position	Primer Length	Sequence (5'-3')	Reference
VP3-VP1-2A	92378	Sense	2060-2084[a]	25	ATGITIGGIACICAYGTNGTNTGGG	(1)
	187	Sense	2435-2454[a]	20	ACIGCIGYIGARACIGGNCA	(2)
	VP1F	Sense	2645-2669[a]	25	GARATGGCICARATYAGRIGIAAAT	(3)
	VP1F78	Sense	2650-2674[a]	25	GAGATGGCCCAGATTAGAAGAAAG	(3)
	VP1FB	Sense	2690-2712[a]	23	TATGTIAGRTTTGAYTCWGARTA	(3)
	VP1FA	Sense	2766-2787[a]	22	TW GTIATGCARTAYATGT AT GT	(3)
	92379	Antisense	2768-2790[a]	23	GGIGCICCIGGIGGIGGNACATACAT	(1)
	VP1R	Antisense	3077-3100[a]	24	IGCYCTIGGIGGICKICCRCACCA	(3)
	PRPP	Sense	3086-3108[a]	23	TGGTGYCCIMGISCICCIMGTGC	(3)
	92383	Antisense	3498-3518[a]	21	CCICCICAITCWCCWGGTTC	(1)
VP1	92380	Sense	2645-2669[b]	25	GAI ATG GTICAIATYAGR AGRAAA T	(1)
	92580	Sense	2435-2454[b]	20	ACI GCI GYI GAR ACI GGN CA	(2)
VP1(HRV-A)	W.F	Sense	1995-2017[c]	23	MGHTTYAGYTTYATGTTYTGTGG	(4)
	X.F	Sense	2430-2448[c]	19	TRGAYGCWGCWGARACWGG	(4)
	X.R	Antisense	3333-3358[c]	26	GTRTTTGTKCGGTADATGAYTARRTC	(4)
	W.R	Antisense	3525-3547[c]	23	CCACARTCWCCWGGYTCACADGG	(4)
VP4-VP2[d]	Y.F	Sense	458-478[c]	21	CCGGCCCCTGAATGYGGCTAA	(4)
	Z.F	Sense	547-569[c]	23	ACCRACTACTTTGGGTGTCCGTG	(4)
	Z.R	Antisense	1087-1109[c]	23	TCWGGHARYTTCCAMCACCANCC	(4)
	Y.R	Antisense	1125-1149[c]	25	ACATRTTYTSNCCAAANAYDCCCAT	(4)

[a] The primer positions were numbered according to the complete HRV14 genome sequence (5).
[b] The primer positions were numbered according to HRV1B complete genome sequence (6).
[c] the 5’ base position was numbered in accordance with the HRV-B serotype 14 genome (GenBank accession number NC_001490).
[d] these primers targets both human rhinovirus (HRV) and human enterovirus (HEV).
Abbreviations for the variable nucleotide (letters) in the sequence column: “I”, Inosine; “K”, G or T; “R”, A or G; “Y”, C or T; “M”, C or A; “W”, A or T; “V”, A or C or G; “S”, G or C; “N”, A or C or G or T.

Step 1

Pre-PCR: Using SuperScript™ III Platinum™ One-Step RT-qPCR Kit, the following amounts were used per each reaction: 12.5 µl of 2x reaction mix, 0.25 µl for each primers (10µM), 1 µl of Superscript" III RT/Platinum" TaqMix, 1 µl $MgSO_4$ 50mM, and 6 µl nuclease free wate. Then 5 µl of the RNA template was added,(total of 25 µl), using master mixes A to F were used.

PCR Thermal Cycling: the mixture was subjected to 50°C for 30 min reverse transcription, 95°C, 3 min pre-denaturation, 95°C, 30s denaturation, 42°C, 30s primer annealing, and then 60°C, 1 min extension.

Gel-Check: using 1% agarose E-Gel® (Invitrogen™, Thermo Fisher Scientific, UK). 4µL E-Gel® Sample Loading Buffer, 1X (Invitrogen™, UK) was mixed with 10µL of PCR product and loaded in the E gel well. 20 µL of 100 bp DNA ladder (Invitrogen™, UK) was used as the size marker

Step 2

Pre-PCR: The same as step 1, but the modifications are: 0.5 for each forward and reverse primers (10µM), 5.5 µl nuclease free water, and without any additional $MgSO_4$

PCR Thermal Cycling: the same as step 1

Gel Check: the same as step 1

Step 3

Using real-time qPCR LightCycler 480 (Roche) to detect, and quantify the HRV load (double-check).

Step 4

Using **a different kit**; SuperScript™ III Platinum™ One-Step RT-PCR Kit (Invitrogen, thermo fisher, United Kingdom), and repeat steps 1 and 2

Step 5

Nested and semi-nested PCR:using the PCR products of A, B, and C with D, E, and E, respectively.

Step 6

Heating the template before adding it to the master mix to make sure we get the straight template rather than the folding form.

Step 7

Increasing the temperature of the RT-PCR (60 instead of 50).

Step 8

- **Pre-PCR**: the same as step 2, with variable amount of Mg and nuclease free water (Mg titration).

Step 9

- **PCR Thermal Cycling**: the same as step 1, but using 68°C extension instead of 60°C

Step 10

- **Gel-Check**: the same as step 1

FIGURE S1. Stage A in PCR optimisation.

This flow chart summarises the PCR optimisation steps using DST-purified primers targeting VP3/VP1 and VP1 in the HRV region. RT-qPCR: reverse-transcriptase quantitative polymerase chain reaction; VP: viral-capsid protein.

TABLE S2. Master mixes and primer combinations were used to detect the variable regions of the HRV genome.

Master mix	Primers	PCR product length (bp)	1st and/or 2nd round PCR use(s)
A	F 92378<br>R 92379	730	1st PCR
B	92580 = F187<br>R 92383	1100	1st PCR
C	F 92380<br>R 92383	873	1st PCR
C.2	*F VP1F*<br>*R 92383*	873	1st PCR
D	F 92580 = F 187<br>R 92379	355	1st PCR & 2nd PCR
E	F VP1F<br>R VP1R	455	1st PCR & 2nd PCR
F	F PRPP<br>R 92383	432	1st PCR
W	W.F<br>W.R	1530	1st PCR
X	X.F<br>X.R	929	2nd PCR
Y	Y.F<br>Y.R	692	1st PCR
Z	Z.F<br>Z.R	563	2nd PCR

TABLE S3. The preparation of the first-round PCR master mixes.

	**Component**	**Amount for 1 reaction (in µL)**
1.	2X Reaction Mix[1]	12.5
2	Sense primer (10 µM)	0.5
3	Anti-sense primer (10 µM)	0.5
4	Superscript” III RT/Platinum” TaqMix	1
5	$MgSO_4$	-
6	Nuclease-free water	5.5
	**The total volume of master mix**	20 µL

[1] The Superscript III One-step RT-PCR kit contains the 2x Reaction Mix with 0.4 mM of each dNTP and 3.2 mM $MgSO_4$ as active ingredients.

TABLE S4. The preparation of the second-round PCR reactions using the LightCycler® Multiplex DNA master mix.

	component	The amount for one reaction (in µL)
1	5X Reaction Mix	5
2	Sense primer (10 µM)	1
3	Anti-sense primer (10 µM)	1
4	Nuclease-free water	17
	**The total volume of the master mix**	24 µL

TABLE S5. The preparation of the second-round PCR reactions using the Platinum® SYBR® Green qPCR SuperMix-UDG.

	component	The amount for one reaction (in µL)
1	Platinum® SYBR® Green qPCR SuperMix-UDG	12.5
2	Sense primer (10 µM)	1
3	Anti-sense primer (10 µM)	1
4	Nuclease-free water	9.5
	**The total volume of the master mix**	24 µL

TABLE S6. Thermal Cycling conditions used in PCR reactions.

**Steps**	**cDNA synthesis & pre-denaturation**		**Denature**	**Anneal**	**Extend**	**Final extension (optional)**
**No. of cycles**	1 cycle		40 cycles			1 cycle
**Temperature**	45°C	95°C	95°C	42°C	68°C	68°C
**Time**	30 min	3 min	30 s	30 s	1 min	5 min

TABLE S7. Reagents used in sequencing reactions.

Reagent	Volume
Sequencing primer (1µM in TE 0.1M)	1.6µl
Big Dye Reaction v3.1 (ABI Part No. 4336911)	0.16µl
ddH_2O	4.84µl
**Total volume of master mix**	8.6µl
PCR product	1.4µl

TABLE S8. Cycling conditions used in sequencing reactions.

Number of cycles	Temperature	Time
1	96°C	20s
25	96°C	10s
	50°C	5s
	60°C	4 min
	4ºC soak until required	

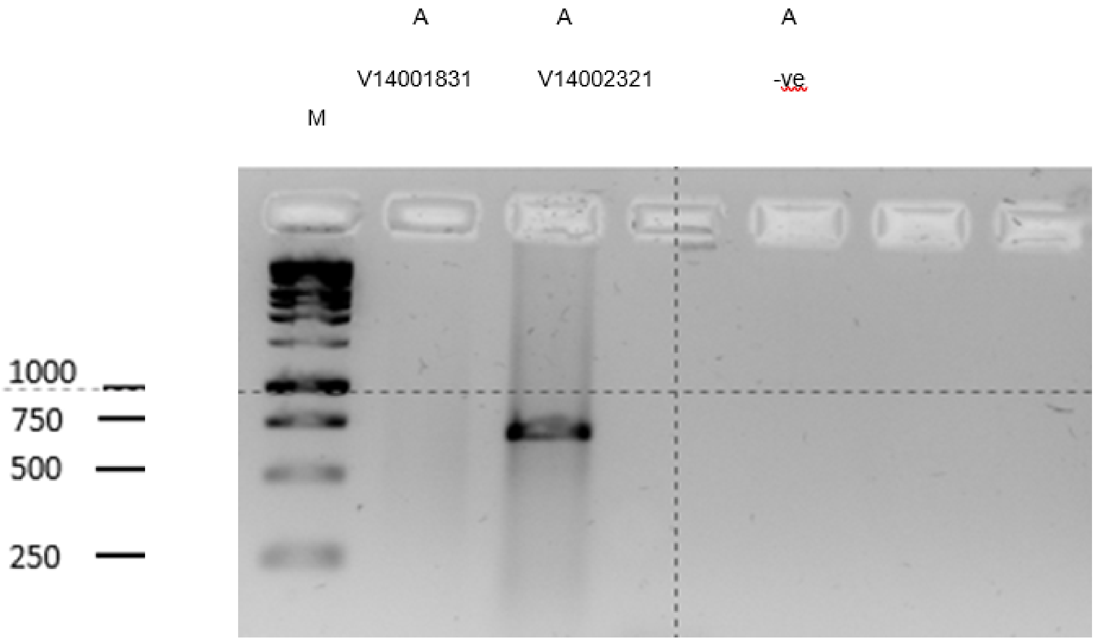

FIGURE S2. PCR product representation of master mix A.

This gel picture is showing a single, brighter product band on the 1 % agarose gel, of the right size (~730 bp) with the sample V14002321, but not with sample V14001831 although both samples have almost the same HRV load (the same C_t value), and this reflects the high variation in the targeted region between HRV species. The "A" assay (master mix) is targeting a highly variable region of VP3/VP1 in HRV. GeneRuler 1 kb DNA Ladder (Thermo Scientific, UK) was used.

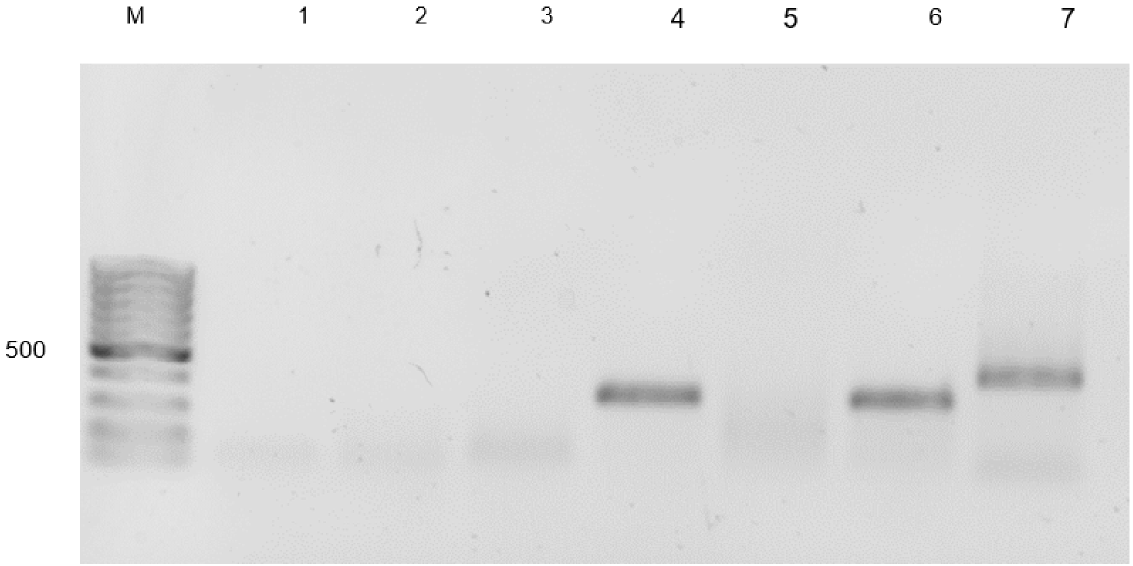

FIGURE S3. The second-round PCR product representation of A:D and C:E assays.

Using 1% agarose gel, this figure showing a product of ~355 bp, and ~455 bp for A:D and C:E assays, respectively. the Platinum® SYBR® Green qPCR SuperMix-UDG (Thermo Fisher, cat. no. 11733038) was used to prepare D and E master mixes. M: GeneRulerTM 100 bp DNA Ladder (marker) (Thermo Scientific, UK), Lane 1: A:D with V14000746 specimen, Lane 2: A:D with V14001545 specimen, Lane 3: A:D with V14001831 specimen, Lane 4: A:D with V14002321

specimen, Lane 5 is a replicate of lane 3 (both gave no product), Lane 6 is a replicate for lane 4 (both gave the same positive product with the same length), Lane 7: C:E with V14002321 specimen.

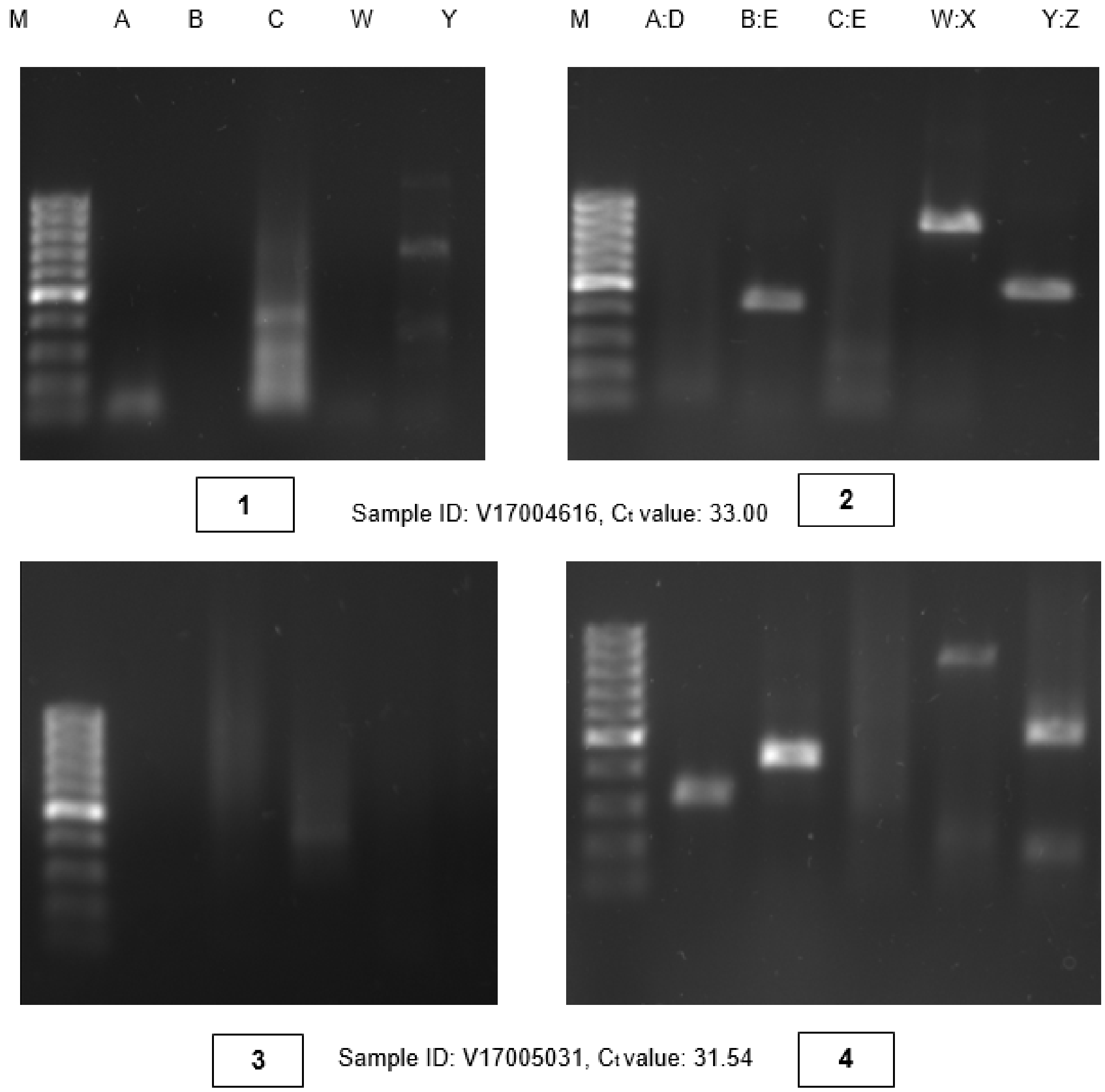

FIGURE S4. Gel electrophoresis of first-round and second-round PCR products of 10 different assays.

The representative 1 % agarose gel pictures show PCR amplification products for 5 different first-round assays (pictures 1 and 3) and 5 related second-round PCR assays (pictures 2 and 4) for two different specimens with the corresponding HRV C_t value. Figures 2 and 4 show a product of ~455 bp, ~929 bp, and ~563 bp for B:E, W:X, and Y:Z assays, respectively for the two different respiratory samples, in addition to ~355 bp for A:D assay for the second sample (picture 4). M, GeneRuler™ 100 bp DNA marker.

TABLE S9. The PCR product positivity rate of HRV-VP assays using the fifteen respiratory samples.

Assay	Assay's target	Positivity rate
**A**	VP3/VP1	6/15 (40 %)
**B**	VP1	1/15 (7 %)
**C**	VP1	2/15 (13 %)
**W**	VP1	0/15 (0 %)
**Y**	VP4/VP2	8/15 (53 %)
**A:D**	VP1	6/15 (40 %)
**B:E**	VP1	5/15 (33 %)
**C:E**	VP1	3/15 (20 %)
**W:X**	VP1	4/15 (27 %)
**Y:Z**	VP4/VP2	13/15 (87 %)

TABLE S10. The successful representation of the Sanger sequencing primers.

Primer	Primer Failure No.	Total reactions
F92378	0	6
R92379	7	25
F92580	4	13
Y.F	0	7
Y.R	0	7
F VP1F	0	6
VP1R	1	6
X.F	3	6
X.R	2	6
Z.F	5	22
Z.R	2	15
Total	24	119

TABLE S11. A summary of gel-check, sequencing successful, and sequences assembly results of the clinical HRV tested samples.

Specimen ID	C_t value	PCR product on gel (+, -)						Total successful seq. ratio	Contigs no.		Contig length*	No. of seq.	No. (top, bottom)	Avg Coverage
		A	Y	A:D	B:E	W:X	Y:Z							
V14002321	18.06	+	+	+	+	-	+	14/16	2	Contig 1	671	4	(2,2)	2.93
										Contig 2	686	5	(3,2)	2.22
V14001831	18.10	-	+	+	+	-	+	7/7	1	Contig 1	618	5	(3,2)	3.06
V14001545	19.08	+	+	-	-	-	+	6/8	1	Contig 1	643	4	(2,2)	2.83
V14000746	19.12	+	-	+	+	-	+	11/16	3	Contig 1	465	3	(1,2)	2.01
										Contig 2	703	4	(2,2)	2.26
										Contig 3	574	3	(1,2)	2.46
V17004616	33.00	-	+	-	+	+	+	9/12	1	Contig 1	62	2	(1,1)	1.76
V17004470	31.10	-	-	-	-	+	+	3/5	0	-	-	-	-	-
V17005728	26.69	+	+	+	-	+	+	12/13	2	Contig 1	634	2	(1,1)	1.49
										Contig 2	600	5	(3,2)	2.95
V17006129	31.90	+	+	-	-	-	+	4/5	1	Contig 1	665	2	(1,1)	1.91
V17006131	29.91	+	-	-	-	-	-	6/10	0	-	-	-	-	-
V17006286	33.07	-	+	+	-	-	+	3/3	0	-	-	-	-	-
V17004189	28.88	-	-	+	-	-	+	2/3	0	-	-	-	-	-
V17003665	30.09	-	-	-	-	-	+	2/2	0	-	-	-	-	-
V17004870	21.09	-	+	-	-	-	-	4/7	1	Contig 1	229	2	(1,1)	1.53
V17005031	31.54	-	-	+	+	+	+	5/8	0	-	-	-	-	-
V17004381	28.75	-	-	-	-	-	+	2/2	0	-	-	-	-	-

*All without gaps

Pro Assembly algorithm parameters used for contigs assembly using SeqMan Pro (DNASTAR Lasergene version 15) are as the following: match size = 25, minimum match percentage = 70, minimum sequence length = 50, gap penalty = 0.00, gap length penalty = 0.00, match spacing = 150, maximum mismatch end bases = 15.

TABLE S12. Description of the VP regions' sequences assembly.

No.	Reaction name	Pre-trim length	Trimmed length	Sequence range	Contig	Average quality
			**Sample V14002321**			
1.	11-V14002321-Y_ZF	647	541	(1>541)	Contig 1	30
2.	20-V14002321-AD_F92580	283	34	(24>57)	-	55
3.	20-V14002321-AD_R92379	326	68	(1>68)	Contig 2	19
4.	32-V14002321-WX_R92379	144	72	(16>87)	Contig 2	18
5.	45-V14002321-YZ_ZF	320	39	(28>66)	Contig 1	14
6.	45-V14002321-YZ	473	153	(1>153)	Contig 1	26
7.	V14002321-A_F92580	326	67	(3>69)	Contig 2	23
8.	4-V14002321-A_F92378	694	682	(1>682)	Contig 2	27
9.	4-V14002321-A_R92379	698	603	(1>603)	-	35
10.	11-V14002321-Y_YF	895	624	(1>624)	-	32
11.	11-V14002321-Y_YR	638	629	(4>632)	Contig 1	38
12	32-V14002321-WX_XR	203	26	(22>47)	-	23
13.	25-V14002321-BE_FVP1F	386	75	(34>108)	-	14
14.	25-V14002321-BE_VP1R	411	92	(38>129)	-	12
	**Average**	**460**	**265 (57%)**			**27**
			**Sample V14001831**			
1.	12-V14001831-Y_ZF	670	502	(1>502)	Contig 1	28
2.	26-V14001831-BE_FVP1F	427	160	(23>182)	-	17
3.	26-V14001831-BE_R92379	426	219	(33>251)	-	10
4.	46-V14001831-YZ_ZF	280	57	(30>86)	Contig 1	38
5.	46-V14001831-YZ_ZR	436	108	(1>108)	Contig 1	27
6.	12-V14001831-Y_YF	722	598	(1>598)	Contig 1	21
7.	12-V14001831-Y_YR	682	610	(2>611)	Contig 1	26
	**Average**	520	322 (62%)			**24**
			**Sample V14001545**			
1.	13-V14001545-Y_YF	794	610	(1>610)	Contig 1	30
2.	13-V14001545-Y_YR	631	618	(1>618)	Contig 1	38
3.	13-V14001545-Y_ZF	544	500	(1>500)	Contig 1	26
4.	47-V14001545-YZ_ZR	398	72	(11>82)	Contig 1	22
5.	5-V14001545-A_F92378	408	286	(32>317)	-	17
6.	5-V14001545-A_R92379	362	332	(31>362)	-	16
	**Average**	**523**	**403 (77%)**			**25**
			**Sample V14000746**			
1.	14-V14000746-Y_YF	1000	548	(23>570)	Contig 3	30
2.	14-V14000746-Y_YR	641	493	(12>504)	Contig 3	25
3.	14-V14000746-Y_ZF	754	360	(1>360)	Contig 3	30
4.	21-V14000746-AD_F92580	463	321	(1>321)	Contig 2	47
5.	21-V14000746-AD_R92379	516	315	(2>316)	Contig 2	40
6.	27-V14000746-BE_FVP1F	907	417	(3>419)	Contig 1	39
7.	27-V14000746-BE_VP1R	628	418	(1>418)	Contig 1	37
8.	48-V14000746-YZ_ZR	276	68	(1>68)	-	16
9.	V14000746-BE_R92379	424	93	(22>114)	Contig 1	25
10.	6-V14000746-A_F92378	696	673	(2>674)	Contig 2	26
11.	6-V14000746-A_R92379	673	267	(26>292)	Contig 2	26
	**Average**	634	361 (57%)			31
			**Sample V17004616**			
1.	7-V17004616-Y_ZF	694	524	(36>559)	-	24
2.	22-V17004616-BE_R92379	438	394	(22>415)	-	15
3.	22-V17004616-BE_VP1R	239	48	(20>67)	-	10
4.	28-V17004616-WX_R92379	131	52	(20>71)	Contig 1	14
5.	28-V17004616-WX_XF	245	17	(25>41)	-	15
6.	34-V17004616-YZ_ZR	189	64	(1>64)	-	29
7.	7-V17004616-Y_YF	316	41	(27>67)	-	10
8.	7-V17004616-Y_YR	665	116	(23>138)	-	63
9	22-V17004616-BE_FVP1F	353	53	(20>72)	Contig 1	18
	**Average**	363	146 (40%)			22
			**Sample V17004470**			
1.	29-V17004470-WX_XR	135	9	(42>50)	-	23
2.	35-V17004470-YZ_ZR	154	69	(1>69)	-	26
3.	29-V17004470-WX_XF	211	81	(15>95)	-	17
	**Average**	167	53 (32%)			**22**
			**Sample V17005728**			

1.	8-V17005728-Y_YF	882	595	(1>595)	Contig 2	29
2.	8-V17005728-Y_YR	840	508	(1>508)	Contig 2	23
3.	8-V17005728-Y_ZF	499	487	(2>488)	Contig 2	26
4.	15-V17005728-AD_R92379	287	59	(13>71)	-	13
5.	30-V17005728-WX_R92379	169	68	(20>87)	-	27
6.	36_V17005728-YZ_ZF	315	75	(21>95)	Contig 2	16
7.	36-V17005728-YZ_ZR	235	84	(5>88)	Contig 2	18
8	1-V17005728-A_F92378	645	632	(1>632)	Contig 1	32
9	1-V17005728-A_R92379	687	307	(2>308)	Contig 1	27
10	V17005728-A_F92580	586	336	(111>446)	-	14
11.	30-V17005728-WX_XF	167	54	(25>78)	-	15
12.	30-V17005728-WX_XR	346	57	(25>81)	-	24
	**Average**	**472**	**272 (58%)**			**22**
	**Sample V17006129**					
1.	2-V17006129-A_R92379	634	634	(1>634)	Contig 1	32
2.	37-V17006129-YZ_ZR	291	115	(3>117)	-	23
3.	2-V17006129-A_F92378	637	626	(1>626)	Contig 1	30
4.	2-V17006129-A_F92580	290	183	(7>189)	-	15
	**Average**	**463**	**390 (85%)**			**25**
	**Sample V17006131**					
1.	3-V17006131-A_F92580	163	136	(28>163)	-	17
2.	9-V17006131-Y_YF	704	265	(19>283)	-	29
3..	9-V17006131-Y_YR	661	157	(17>173)	-	18
4.	9-V17006131-Y_ZF	519	466	(1>466)	-	28
5.	3-V17006131-A_F92378	640	286	(162>447)	-	17
6.	16-V17006131-AD_F92580	245	25	(28>52)	-	33
	**Average**	**489**	**223 (46%)**			**24**
	**Sample V17006286**					
1.	39-V17006286-YZ_ZF	213	8	(32>39)	-	73
2.	17-V17006286-AD_F92580	153	19	(15>33)	-	17
3.	17-V17006286-AD_R92379	156	47	(4>50)	-	15
	**Average**	**174**	**25 (14%)**			**35**
	**Sample V17004189**					
1.	40-V17004189-YZ_ZR	20	20	(61>80)	-	9
2.	18-V17004189-AD_R92379	46	46	(25>70)	-	28
	**Average**	**33**	**33 (100%)**			**19**
	**Sample V17003665**					
1.	41-V17003665-YZ_ZF	159	8	(17>24)	-	21
2.	41-V17003665-YZ_ZR	148	45	(25>69)	-	10
	**Average**	**154**	**27 (18%)**			**16**
	**Sample V17004870**					
1.	10-V17004870-Y_YR	320	176	(7>182)	Contig 1	31
2.	10-V17004870-Y_ZF	285	208	(17>224)	-	15
3.	10-V17004870-Y_YF	291	168	(19>186)	Contig 1	15
4.	23-V17004870-BE_FVP1F	139	52	(34>85)	-	12
	**Average**	**259**	**151 (59%)**			**19**
	**Sample V17005031**					
1.	24-V17005031-BE_FVP1F	243	47	(25>71)		27
2.	24-V17005031-BE_R92379	167	74	(94>167)		10
3.	24-V17005031-BE_VP1R	310	61	(20>80)		10
4.	43-V17005031-YZ_ZR	198	59	(1>59)		14
5.	19-V17005031-AD_F92580	159	57	(5>61)		15
	**Average**	**215**	**60 (28%)**			**16**
	**Sample V17004381**					
1.	44-V17004381-YZ_ZF	139	31	(45>75)	-	42
2.	44-V17004381-YZ_ZR	246	99	(6>104)	-	21
	**Average**	**193**	**65**			**32**

Trim ends parameters used: trace threshold = 8, quality threshold = 10, non-trace window size = 30.

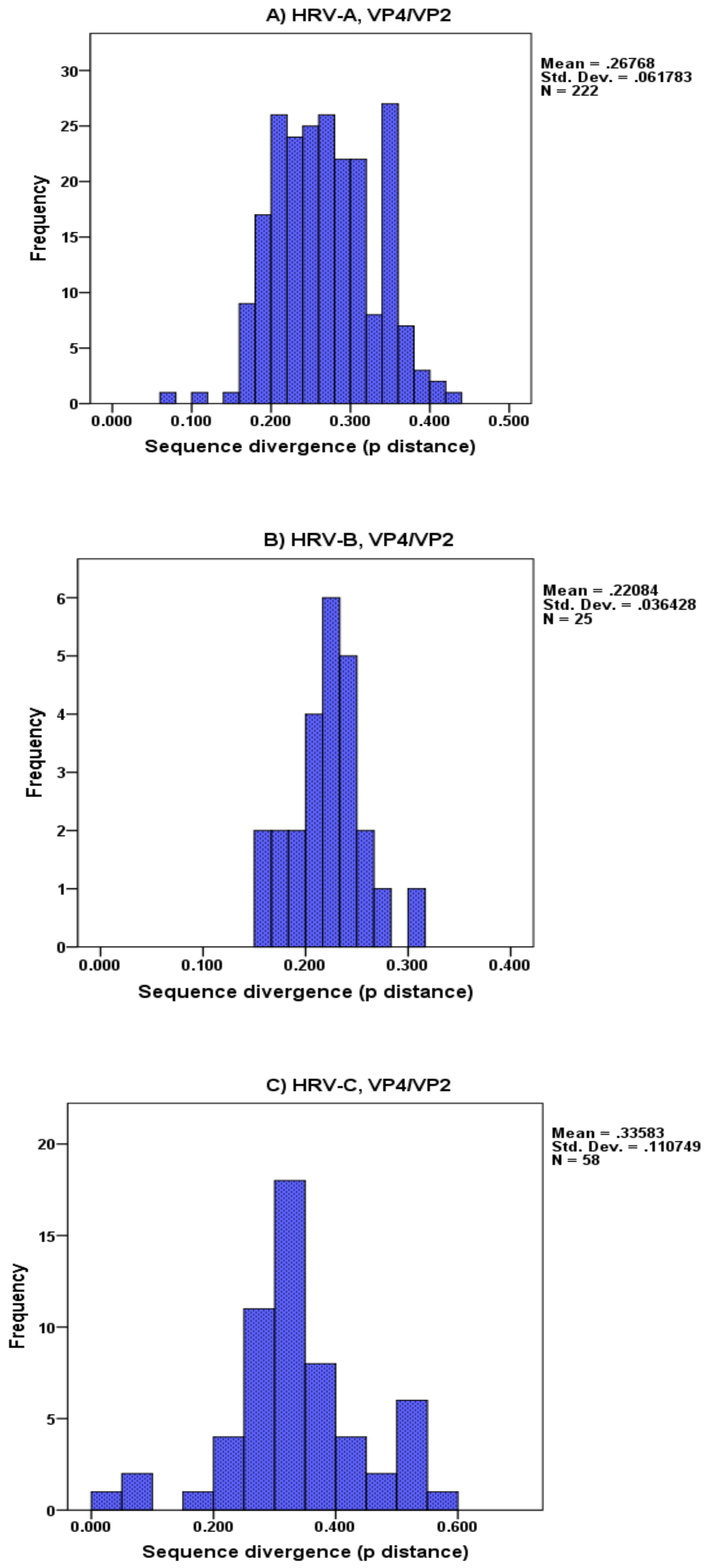

FIGURE S5. Distribution of nucleotide p-distances for HRV-A (A), HRV-B (B), and HRV-C (C) detected in the study compared with sequences from reference strains based on the nucleotide sequences of the VP4/VP2 region.

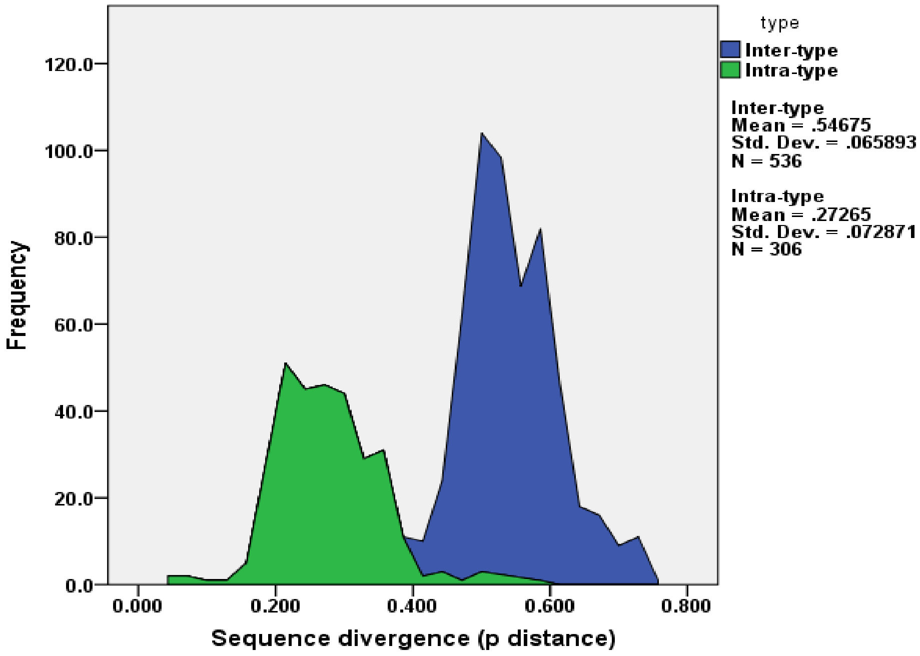

FIGURE S6. Distribution of pairwise nucleotide p-distances for the VP4/VP2 region of all HRV sequences.

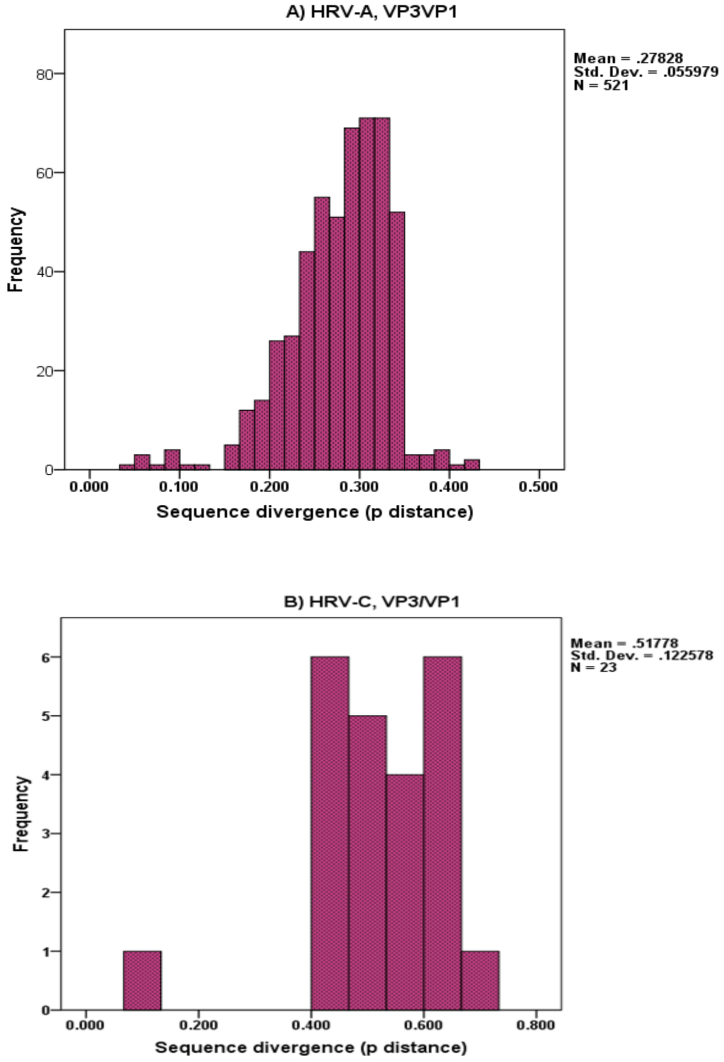

FIGURE S7. Distribution of nucleotide p-distances for HRV-A (A), and HRV-C (B) detected in the study compared with sequences from reference strains based on the nucleotide sequences of the VP3/VP1 region.

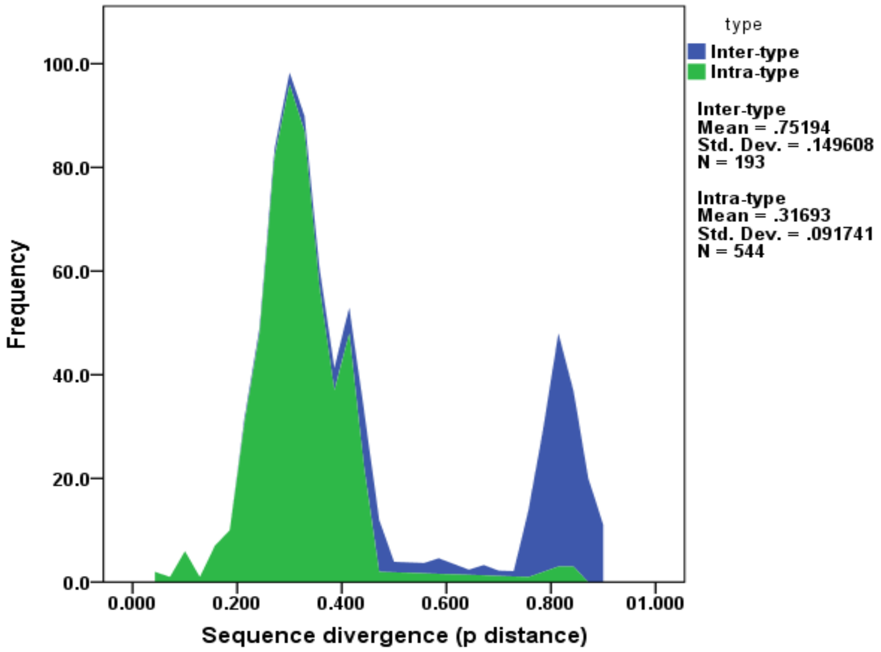

FIGURE S8. Distribution of pairwise nucleotide p-distances for the VP3/VP1 region of all HRV sequences.

TABLE S13. The multiple sequence alignments (MSAs) data and genotype identification.

MSA no.	VP region	MSA length (bps)	Samples	Genotype	Bootstrap	Distance
MSA 1	VP4/VP2	512	V14001831_Contig_1	HRV-C_32	100	0.047
			V17005728_Contig_2	HRV-C_45	73	0.289
MSA 2	VP4/VP2	545	V14002321_Contig_1	HRV-A_21	100	0.065
			V14001831_Contig_1	HRV-C_32	100	0.052
			V14001545_Contig_1	HRV-B_79	55	0.150
			V14000746_Contig_3	HRV-A_1	N	0.196
MSA 3	VP4/VP2	142	R_41-V17003665-YZ_ZR	HRV-C_24	69	0.268
MSA 4	VP4/VP2	427	V17006131-Y_ZF	HRV-C_45	N	0.296
			V17004870-Y_ZF	HRV-C_32	N	0.927
MSA 5	VP4/VP2	310	V14002321-YZ_ZF	HRV-A_21	95	0.104
			V17006286-YZ_ZF	HRV-A_50	50	0.264
MSA 6	VP4/VP2	177	R_V17004616-YZ_ZR	HRV-A_98	N	0.173
			R_V14000746-YZ_ZR	HRV-A_1	N	0.211
			R_V17004470-YZ_ZR	HRV-A_13	N	0.236
MSA 7	VP4/VP2	189	R_V17006129-YZ_ZR	HRV-C_3	79	0.181
			R_V17004381-YZ_ZR	HRV-C_36	100	0.066
MSA 8	VP4/VP2	170	V17004870-Y_ZF	HRV-C	99	0.476
MSA 9	VP3/VP1	478	V14002321_Contig_2	HRV-A_21	99	0.096
			V14000746_Contig_2	HRV-A_40	99	0.089
			17005728_Contig_1	HRV-C_7	99	0.506
			V17006129_Contig_1	HRV-C_7	99	0.098
MSA 10	VP3/VP1	335	V14002321_Contig_2	HRV-A_21	97	0.046
			V14002321-AD_F92580	HRV-A_45	86	1.883
			V14000746_Contig_2	HRV-A_40	96	0.055
			V17005031-AD_F92580	HRV-C_7	86	2.191
MSA 11	VP3/VP1	81	V14002321-AD_F92580	HRV-A_21	80	0.120
			V17005031-AD_F92580	HRV-A_47	52	0.087
MSA 12	VP3/VP1	104	R_V14001545_A_R92379	HRV-A_40	63	0.112
			R_V17005728-WX_R92379	HRV-A_78	83	0.133
MSA 13	VP3/VP1	101	V14002321_Contig_2	HRV-A_21	99	0.053
			R_V17005728-WX_R92379	HRV-A_78	82	0.133
MSA 14	VP3/VP1	261	V14002321_Contig_2	HRV-A_21	99	0.072
			V17004616-WX_XF	HRV-A_59	58	0.208
			V17004470-WX_XF	HRV-A_41	N	0.634
MSA 15	VP3/VP1	364	V14002321_Contig_2	HRV-A_21	100	0.059
			R_V14001545_A_R92379	HRV-A_21	100	0.179
MSA 16	VP3/VP1	410	V14000746_Contig_1	HRV-A_21	99	0.114
			R_V14001831_BE_R92379	HRV-A_21	99	0.118

MSA: Multiple Sequence Alignment, N: not significant bootstrap (< 50%).

TABLE S14. A summary of recombination events detected in the multiple sequence alignments.

MSA no.	Overall Rec. no.	Study Sample	Major Parent	Minor Parent	R	B	M	C	S	T
MSA 1	6	-	-	-	-	-	-	-	-	-
MSA 2	6	V14000746_Contig_3	A_11	B_37	$2.18x10^{-7}$	$1.30x10^{-2}$	$1.16x10^{-3}$	$1.71x10^{-4}$	$7.08x10^{-3}$	$7.91x10^{-4}$
MSA 3	-	-	-	-	-	-	-	-	-	-
MSA 4	1	-	-	-	-	-	-	-	-	-
MSA 5	2	V17006286-YZ_ZF	A_50	Unknown	$1.64x10^{-3}$	-	-	-	-	$1.24x10^{-3}$
MSA 6	1	R_V17004470-YZ_ZR	Unknown	A_19	$3.80x10^{-2}$	-	-	-	-	-
MSA 7	-	-	-	-	-	-	-	-	-	-
MSA 8	-	-	-	-	-	-	-	-	-	-
MSA 9	1	-	-	-	-	-	-	-	-	-
MSA 10	1	-	-	-	-	-	-	-	-	-
MSA 11	-	-	-	-	-	-	-	-	-	-
MSA 12	-	-	-	-	-	-	-	-	-	-
MSA 13	-	-	-	-	-	-	-	-	-	-
MSA 14	1	V17004616-WX_XF	A_100	Unknown	$1.71x10^{-2}$	-	$4.93 x10^{-2}$	-	-	$2.88 x10^{-2}$
MSA 15	-	-	-	-	-	-	-	-	-	-
MSA 16	-	-	-	-	-	-	-	-	-	-

The recombination detection programmes used: RDP (R), Bootscan (B), Maximum X2 (M), Chimaera (C), SiScan (S) and 3Seq (T).

The recombination events were assessed by the average p values of recombination events with <1.00 x10-5 considered significant. None of the recombinant sequences was detected by more than two programmes with the average p values of recombination events of < 1.00 x10-5.

Abbreviations: MSA: multiple sequence alignment; Rec: recombination.

References

1. Vlasak M, Blomqvist S, Hovi T, Hewat E, Blaas D. Sequence and structure of human rhinoviruses reveal the basis of receptor discrimination. Journal of virology. 2003;77(12):6923-30.
2. Oberste MS, Maher K, Flemister MR, Marchetti G, Kilpatrick DR, Pallansch MA. Comparison of classic and molecular approaches for the identification of untypeable enteroviruses. Journal of clinical microbiology. 2000;38(3):1170-4.
3. Laine P, Savolainen C, Blomqvist S, Hovi T. Phylogenetic analysis of human rhinovirus capsid protein VP1 and 2A protease coding sequences confirms shared genus-like relationships with human enteroviruses. Journal of general virology. 2005;86(3):697-706.
4. Wisdom A, Leitch EM, Gaunt E, Harvala H, Simmonds P. Screening respiratory samples for detection of human rhinoviruses (HRVs) and enteroviruses: comprehensive VP4-VP2 typing reveals high incidence and genetic diversity of HRV species C. Journal of clinical microbiology. 2009;47(12):3958-67.
5. Lee W-M, Monroe S, Rueckert R. Role of maturation cleavage in infectivity of picornaviruses: activation of an infectosome. Journal of virology. 1993;67(4):2110-22.

6. Hughes PJ, North C, Jellis CH, Minor PD, Stanway G. The nucleotide sequence of human rhinovirus 1B: molecular relationships within the rhinovirus genus. Journal of general virology. 1988;69(1):49-58.
